# The Impact of Broadband Infrastructure Construction on Medical Resource Mismatch: Quasi-Natural Experiment From the Broadband China Policy

**DOI:** 10.2196/53921

**Published:** 2024-03-21

**Authors:** Yulin Chai, Xiaoping Yuan, Lin Guo, Zhongming Chen

**Affiliations:** 1 School of Management Shandong Second Medical University Weifang China; 2 School of Psychology Shandong Second Medical University Weifang China

**Keywords:** broadband, infrastructure, medical resources, resource mismatch, Broadband China Policy

## Abstract

**Background:**

Whether the construction of broadband infrastructure can alleviate the problem of mismatched medical resources is crucial to the national information strategy, residents’ well-being, and social equity. However, the academic community lacks a comprehensive theoretical analysis and rigorous empirical research on this issue.

**Objective:**

This study aims to construct a preliminary theoretical framework to scientifically assess the effects of broadband infrastructure development on the mitigation of health care resource mismatch from both theoretical and empirical perspectives, to explore the potential mechanisms of influence, and ultimately to develop several practical policy recommendations.

**Methods:**

We first used a theoretical analysis to propose testable theoretical hypotheses and establish a preliminary theoretical framework. Then, based on balanced panel data from 300 cities from 2010 to 2021, a 2-way fixed effects difference-in-differences model was used for empirical testing. Mechanism tests, robustness analyses, and heterogeneity analyses were further conducted.

**Results:**

The research findings demonstrate that the Broadband China Policy significantly reduces the degree of mismatch in medical resources by primarily using innovation effects and integration effects, resulting in a reduction of 13.2%. In addition, the heterogeneity analysis reveals that the central and eastern regions, cities with large populations, and areas with a high proportion of young people benefit more significantly.

**Conclusions:**

This study fully confirms, both theoretically and empirically, that broadband infrastructure construction can effectively reduce the mismatch of medical resources not only by expanding the existing literature on the impact of broadband on public services but also by providing valuable opportunities for policy makers to optimize the allocation of medical resources.

## Introduction

### Background

Medical resources encompass disease prevention, treatment, medication supply, and nursing care services. It includes hospitals, pharmaceuticals, and diagnostic resources [1]. The proper allocation of medical resources is crucial for assessing a country’s comprehensive strength. China is experiencing a severe mismatch in medical resources, with uneven distribution among regions, inefficiency in medical services, and a lack of collaboration among medical institutions. The misalignment between medical resources and service providers exacerbates this situation [2,3]. Studies show a substantial geographic mismatch, with Grade 3A hospitals outnumbering primary medical institutions. The severe mismatch hampers social and economic development goals, exacerbates social inequality, and diminishes residents’ well-being [4-6]. Therefore, it is imperative to rectify this mismatch.

Globally, broadband is propelling a new wave of IT development. Many countries prioritize it as a strategic area to enhance national competitiveness. Since 2014, China has been implementing the Broadband China Policy (BCP) to promote broadband and network infrastructure construction. This study examines the impact of broadband infrastructure on medical resource mismatches (MMs), aiming to optimize government policies and improve the mismatch of medical resources.

In theory, the construction of broadband infrastructure can affect the efficiency of medical resource allocation. With the advent of the third industrial revolution, broadband and other network infrastructures have the potential to greatly enhance the mobility of factors, expedite resource processing and integration, and enhance the efficiency of resource allocation [7,8]. The construction of broadband infrastructure can enhance medical resource allocation efficiency and promote the integration of the internet and health care, providing real-time, convenient, and high-quality medical services [9]. In fact, the construction of broadband infrastructure has initially shown results. With the deepening of BCP promotion, the construction rate of provincial-, municipal-, and county-level regional health information platforms in China has reached 100%, 62.8%, and 46.4%, respectively. A total of >7000 public hospitals at or above the secondary level nationwide have access to the regional national health information platform, and >2200 tertiary hospitals have initially achieved interhospital medical service information sharing. Can the construction of broadband infrastructure truly reduce the mismatch in medical resources? What is the real effect, and what are the potential mechanisms? Unfortunately, the academic community has not paid enough attention to these questions. Existing research mainly qualitatively believes that BCP has potential in the medical and health field [10] and can alleviate the shortage of medical resources in remote areas [11] and promote the balanced allocation of medical resources [12]. This study will attempt to scientifically answer these questions from theoretical and empirical perspectives, which can effectively fill the gap in the academic community and have important theoretical value.

To identify the causal relationship between broadband infrastructure construction and mismatched medical resources, there are inherent endogeneity challenges. Broadband infrastructure construction may reduce the problem of mismatched medical resources, whereas regions with low levels of mismatched medical resources may indicate high levels of information technology, improving local broadband infrastructure. Fortunately, in 2013, the State Council issued the *Broadband China* strategy and implementation plan, gradually promoting infrastructure construction. This had a significant exogenous impact on broadband infrastructure levels, with pilot areas having higher levels and nonpilot areas having lower levels.

### Policy Background

Broadband infrastructure is recognized as a strategic public asset that is essential for China’s social development in the contemporary era. It plays a vital role in supporting various sectors and driving global information advancement. Broadband infrastructure, embodied by 5G technology and fiber optics, serves as the underpinning for worldwide digitalization, networking, and intelligent development, with significant potential to reshape industrial patterns and the societal division of labor. The global community, in pursuit of a sustained competitive advantage, has introduced various initiatives for the development of broadband infrastructure. Of note among these efforts are the European Union’s *Connecting Europe Facility—Digital*, aiming to enhance broadband infrastructure coverage across Europe within 3 years; the United States’s *Internet for All* program, designed to deploy broadband infrastructure in underserved areas by 2030 with an investment of US $45 billion; and Japan’s *Digital Garden City Nation*, setting the target of achieving fiber-to-the-home coverage for 99.9% of households by the end of 2027. These endeavors underscore the worldwide recognition of broadband infrastructure as a crucial investment for national progress and development.

In 2013, the State Council of China issued the *Broadband China* strategy and implementation plan [13], which proposed the phased promotion of broadband and other network infrastructure. In 2015, *Guiding Opinions on Accelerating the Construction of High-Speed Broadband Networks and Promoting Network Speed Increase and Fee Reduction* was issued [14]. By the end of 2017, the goals were: 100 Mbps fiber optic access capability in urban areas of prefecture-level cities and most urban areas in nonprefecture-level cities; major cities achieved an average broadband access rate exceeding 30 Mbps and other urban areas reaching 20 Mbps; and comprehensive 4G network coverage in both urban and rural areas, with >80% of rural areas having fiber optic access.

The Ministry of Industry and Information Technology and the National Development and Reform Commission have chosen 120 *Broadband China* demo areas in 3 batches from 2014 to 2016. After selection, local governments will focus on expanding broadband use, increasing network speed and coverage, and advancing infrastructure. According to the construction standards [15], demo cities must meet specific standards. Therefore, the selected cities will reach a leading level in terms of broadband infrastructure construction in the country.

Amid widespread broadband coverage, the evolution of health care information has gained momentum. There has been a continual rollout of specifications for information construction in health care, hastening the integration of health care information systems and the collaborative development of multilevel hospitals. The COVID-19 pandemic has further accelerated the demand for integrated and standardized health care platform construction. Seizing the advantages of broadband infrastructure and driving digitization and information enhancement in health care represents a significant initiative for addressing the mismatch of medical resources. At present, the construction rates for regional medical and health care information platforms stand at 100% for the provincial level, 62.8% for the municipal level, and 46.4% for the county level. Moreover, >7000 public hospitals at the secondary level and above nationwide are linked to the regional population health information platform. In addition, >2200 Grade 3A hospitals have made initial strides in achieving interconnectivity and sharing medical service information within their facilities [16]. The impact of broadband infrastructure construction on mitigating the mismatch of medical resources is yielding tangible outcomes.

### Literature Review

Digital technology has completely transformed health care services, and the application of health information storage and retrieval has grown exponentially [10]. Most public and private medical institutions have announced digitization. The digitalization of medical information can improve profits, reduce waste, enhance the availability of commercial insurance for residents, and improve the efficiency of medical services to reduce residents’ medical expenses [17,18]. Through electronic medical records, remote medical systems, and the Internet of Things, the limitations imposed by resources and location can be reduced, regional disparities can be narrowed, and better disease prevention and resource use can be achieved, thus relieving resource burdens. It also provides an effective platform for comprehensive connectivity and real-time interaction of medical resources, allowing for the rapid and automatic adjustment of rehabilitation strategies and the reconfiguration of medical resources based on specific patient needs [19,20].

Existing research on broadband infrastructure development mainly focuses on 3 aspects. The first is the impact of broadband infrastructure development on innovation. Broadband infrastructure will play an important role in the new round of technological and industrial revolutions by enhancing the level of information technology, promoting digital development, and alleviating resource mismatches to improve innovation capabilities [21,22]. The second is the impact of broadband infrastructure development on the environment. Broadband infrastructure development greatly improves greenhouse gas emission performance, showing significant emission reduction effects, and broadband connectivity promotes the efficiency of green innovation. It can also bring about network spillover effects, which can promote the diffusion and progress of green technologies and further influence green technology innovation [23-25]. The third is the impact of broadband infrastructure development on resource allocation. Broadband infrastructure development can improve resource mismatches, increase resource mobility, thereby reducing the cost of resource allocation, directly or indirectly improving the efficiency of resource allocation, addressing resource mismatches, and addressing spatial spillover effects [26-28]. On the basis of quasi-natural experiments using the BCP, it was found that the BCP has a significant positive impact on resource allocation efficiency. Accelerating the construction and application of broadband infrastructure can optimize decision-making processes and improve resource allocation efficiency. Simultaneously, BCP can enhance the overall level of information infrastructure, reduce resource mismatches, optimize resource allocation, and promote coordinated regional development [29,30].

Several factors contribute to MMs. First, economic development significantly affects resource allocation. Economically weak regions often lack sufficient medical resources, and improvements in income levels have a direct impact on resource availability. This leads to a loss of resources in underdeveloped areas, exacerbating the mismatch. In addition, corruption negatively influences resource allocation [31]. Second, population plays a key role in resource allocation. In remote and sparsely populated areas, the dispersed distribution of the population increases the likelihood of resource mismatches [32]. Moreover, allocating resources solely based on per capita or per area indicators can result in deviations due to higher medical demand among the elderly and children. Finally, factors such as the urbanization rate, financial investment, and per capita gross domestic product (GDP) also influence resource allocation, potentially leading to mismatches [33,34]. In addition, there are 4 unique reasons for MMs in China. These include low physician income and distorted salary systems, an imperfect medical service market hindering resource flow, varying degrees of scarcity of medical resources, and the impact of China’s medical system on resource flow and pricing [35].

In addition, this study offers 3 key contributions. First, it establishes a theoretical framework for the correlation between broadband infrastructure construction and mismatched medical resources, exploring their basic relationship, heterogeneity, and potential mechanisms. This enriches the literature on broadband internet’s impact on public services. Second, it presents empirical evidence from middle- and low-income nations, using authoritative data and rigorous causal strategies to assess the effects of broadband infrastructure on MMs. Finally, drawing from theoretical and empirical analyses, it furnishes theoretical guidance and decision-making support for government bodies to enhance broadband development and resource allocation optimization in the medical sector.

### Theoretical Hypothesis

#### Basic Hypothesis

In the era of IT, broadband is vital to modern society, particularly for BCP’s strategic positioning. By enhancing broadband access and mobile communication, BCP reduces information transmission time, lowers information asymmetry, and boosts public service efficiency [36,37]. Internet health care proved invaluable during the COVID-19 crisis, aiding epidemic control, reshaping physician-patient interactions, and facilitating resource use [38-40]. Information infrastructure development in medical systems alters service patterns, enhancing efficiency [41,42]. The internet empowers patients, tackles inequality, and controls medical costs. In this context, a hypothesis can be proposed:

- Hypothesis 1: BCP can reduce mismatches in the allocation of medical resources.

#### Heterogeneity Hypothesis

China’s current resource allocation model favors the supply side, aggravating regional resource imbalances. Specifically, in health care, most top hospitals are concentrated in high-income regions, concentrating on high-quality medical services [43,44]. Tertiary hospitals, comprising 4% of all hospitals, employ >26% of medical staff and deliver more than 20% of medical services [45]. Eastern coastal regions have abundant medical resources, whereas western areas lack resources, infrastructure, and education [46,47]. Although the BCP has improved nationwide broadband coverage, regional discrepancies persist. Broadband progress is notable in central and eastern regions, narrowing the gap with the West, which still lags behind in key areas [48,49]. From this, we propose the following hypothesis:

- Hypothesis 2: there are differences in the effects of the BCP between different regions, with the central and eastern regions showing greater effects than the western region.

Medical resources in China tend to concentrate in densely populated and economically developed top cities, especially in western regions [46]. The BCP has had a more significant impact on improving medical resource allocation and service quality in top cities than in ordinary prefecture-level cities, with top cities experiencing a 4.2% and 4.8% increase in human and material resources, respectively [50]. Nearly three-quarters of registered internet hospitals in China are concentrated in the top 11 large cities, highlighting their advantages in obtaining medical resources and providing services [51]. Implementing the BCP in top cities can improve local residents’ health status and promote health care services’ accessibility, generating spillover effects on surrounding areas, and promoting public health progress at a macrolevel. Thus, we propose the following hypothesis:

- Hypothesis 3: there are differences in the effects of the BCP between different population sizes, with the effect being greater in cities with larger populations.

In recent decades, global youth populations have faced challenges accessing medical resources and have been neglected in health and social policies. However, the rapid development of digital media and broadband technology presents new opportunities for this group, especially in urban areas with a higher concentration of adolescents [52]. Adolescents are highly active on the internet, with up to 95% using it frequently and having access to computers and tablets at home. This makes the internet an effective platform for adolescent self-care, education, and health-related services [53-55]. In addition, adolescents’ susceptibility to social problems and their increased demand for medical resources and health information further highlight the significance of digital medical platforms and internet health resources in cities with a high proportion of youth populations [56]. Therefore, implementing network medical strategies and resource layouts (BCP) targeted at adolescents in these cities is crucial and more likely to be effective. On the basis of this, we propose the following hypothesis:

- Hypothesis 4: there are differences in the effectiveness of BCP between different population structures, with a greater effect in cities with a higher population of adolescents.

## Methods

### Data Sources

The urban-level data in this study mainly come from the National Bureau of Statistics of China, annual statistical yearbooks of various provinces over the years, annual statistical yearbooks of various cities over the years, annual health statistics yearbooks of China, and reports on the BCP released by the government. Considering data availability and excluding possible impacts from exogenous policy shocks, such as the 2008 global financial crisis and the largest-scale enterprise income tax reform in China, this study ultimately obtained a balanced panel data set covering 300 cities spanning the years 2010 to 2021, with 3600 observations.

### Econometric Equation

In reference to the existing literature [57,58], we constructed the following 2-way fixed effects difference-in-differences (DID) model:



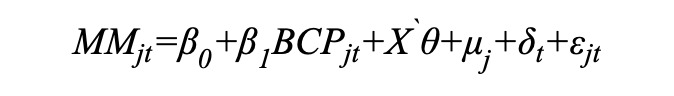



In this equation, *MM_jt_* represents the degree of MM for city j in year t. *BCP_jt_* represents the dummy variable for the BCP*. X`θ* represents a series of city-level control variables to control for other factors’ influence; *μ_j_* represents city fixed effects to control for unobservable factors that vary across cities; *δ_t_* represents year fixed effects to control for unobservable factors that vary over time; *ε_jt_* represents the error term. *β_1_* is the coefficient of interest in this study, and we expect it to be significantly negative. This indicates that the BCP significantly reduces the degree of MM in cities.

### Variable Definition

#### Dependent Variable

The degree of MM in cities is the dependent variable in this study. A previous study defined resource mismatch at the enterprise level [59], stating that optimal resource allocation results in consistent marginal output across different enterprises, thus maximizing total output and efficiency. Conversely, resource mismatch distorts the input-output allocation ratio among different enterprises, leading to a loss in the total output and efficiency. Following their approach, we calculated the degree of MM as follows:



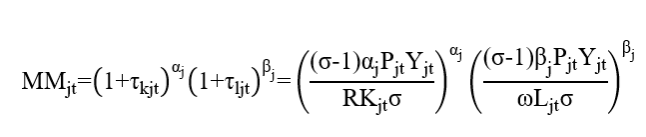



In this equation, τ_kjt_ represents the degree of capital mismatch in city j in year t, where a higher value indicates a greater distortion in the marginal output of capital. τ_ljt_ represents the degree of labor mismatch in city j in year t, where a higher value indicates a greater distortion in the marginal output of labor; α_j_ and β_j_ represent the output elasticities of medical capital and medical labor in cities, respectively, with α_j_+β_j_≠1 [60]; σ represents the elasticity of substitution between outputs of different cities, and this value generally falls between 3 and 10 [61]. To obtain a conservative estimate, in reference to the existing studies [62], we set σ=3; R represents the cost of capital use. In reference to the existing studies [59], we set R as 0.1 in this study; P_jt_Y_jt_ represents city medical expenditure, measured by health expenditure; ωL_jt_ represents the income of medical labor in city j, ω represents the average income in the medical industry within city j, and L represents the number of medical personnel in the city; and K_jt_ represents the amount of medical capital in the city, measured by the total number of hospitals and beds in the city [8]. To facilitate interpretation of the results, we take the logarithm of the calculated results and obtain the dependent variable in this study, the degree of MM in cities.

#### Independent Variable

The core independent variable in this study is the BCP dummy variable. When a city is selected as a pilot city for the BCP in a particular year, the variable is assigned a value of 1 for that year and all subsequent years, whereas it is assigned a value of 0 for other cities. This variable (BCP) serves as the core independent variable in this study.

#### Control Variables

To minimize the potential bias caused by omitted variables, we selected 4 variables—economic level, government intervention, population structure, and urbanization rate—as control variables based on existing studies [26,63,64] and the actual situation.

The first is the economic level (Gdp). Because broadband infrastructure construction is positively correlated with regional economic levels, it is necessary to control for the economic level to identify the impact of broadband infrastructure construction on resource misallocation [65]. This study measures the economic level using *per capita GDP*.

The second is government intervention (Gov). Government intervention is an important factor leading to resource misallocation because the government may suppress the effectiveness of market resource allocation, resulting in market failure. The higher the proportion of fiscal expenditure to regional GDP, the stronger the government’s intervention capacity and thus the higher the level of resource misallocation [66]. This study measures government intervention using the *proportion of government fiscal expenditure to regional GDP*.

The third is the population structure (Pop). The population structure has a significant impact on labor force allocation. The *population dividend* optimizes resource allocation efficiency through the flow of labor, and the unlimited supply of labor offsets the diminishing marginal returns of capital [67]. This study measures population structure using the *average number of employed persons per year divided by the average total population*.

The fourth is the urbanization rate (Urb). The agglomeration effect generated during the process of urbanization can promote the concentration of labor and capital within a region, expand the economic scope and labor market of urban centers and their peripheries, and improve the matching degree between capital, labor, and hospitals, thereby facilitating the flow of resources within the region, enhancing resource allocation efficiency, and reducing resource misallocation [66]. This study measures the urbanization rate using the *proportion of the nonagricultural population to the total population*. The major variables and their definitions are summarized in Table S1 in Multimedia Appendix 1 [59].

### Ethical Considerations

According to China’s Trial Guideline on the Review of Science and Technology Ethics, this study does not collect or use any personal identification information or sensitive data that may harm or disclose personal privacy. The study only focuses on nonhuman entities (cities) and does not involve any direct interaction with individuals or observation of individuals. In addition, this study does not involve any procedures or activities that may raise ethical concerns and complies with all applicable laws and regulations regarding research conduct. Therefore, no ethical approval is required.

## Results

### Descriptive Statistics

Table 1 presents the descriptive statistics for the main variables in this study. The mean of the MM is 1.008, with an SD of 0.360, indicating a significant variation in the misallocation of medical resources among different cities.

**Table 1:** Descriptive statistics (N=3600).

Variables	Values, mean (SD; range)
MM^a^	1.008 (0.360; 0.008-1.520)
BCP^b^	0.212 (0.409; 0.000-1.000)
Gdp^c^	2.481 (1.454; –0.157-5.142)
Gov^d^	0.556 (0.182; 0.218-0.892)
Pop^e^	0.264 (0.120; 0.044-0.472)
Urb^f^	0.452 (0.203; 0.082-0.831)

^a^MM: medical resource mismatch.

^b^BCP: Broadband China Policy.

^c^Gdp: economic level.

^d^Gov: government intervention.

^e^Pop: population structure.

^f^Urb: urbanization rate.

The mean of the BCP is 0.212 (SD 0.409), indicating that 21.22% (764/3600) of the observations in the sample period were classified as the treatment group. The descriptive statistics of the other control variables are generally consistent with those of existing related studies and will not be reiterated here.

### Basic Regression

#### Benchmark Regression Results

To test hypothesis 1, we conduct an empirical test based on equation 1. To demonstrate the effects of different factors, a stepwise regression is used. The specific regression results are presented in Table 2.

**Table 2:** Benchmark regression.

Variables		MM^a^ (column 1)	MM (column 2)	MM (column 3)	MM (column 4)	MM (column 5)
BCP^b^						
	Coefficient (SE)	–0.134 (0.0145)	–0.133 (0.0144)	–0.133 (0.0164)	–0.132 (0.0240)	–0.132 (0.0239)
	*P* value	<.001	<.001	<.001	<.001	<.001
Gdp^c^						
	Coefficient (SE)	N/A^d^	–0.00919 (0.00406)	–0.00931 (0.00407)	–0.00862 (0.00422)	–0.00862 (0.00402)
	*P* value	N/A	.02	.02	.04	.03
Pop^e^						
	Coefficient (SE)	N/A	–0.134 (0.0324)	–0.133 (0.0324)	–0.120 (0.0336)	–0.120 (0.0339)
	*P* value	N/A	<.001	<.001	<.001	<.001
Gov^f^						
	Coefficient (SE)	N/A	0.132 (0.0491)	0.130 (0.0491)	0.133 (0.0506)	0.133 (0.0498)
	*P* value	N/A	.007	.008	.008	.008
Urb^g^						
	Coefficient (SE)	N/A	–0.102 (0.0290)	–0.0976 (0.0290)	–0.0792 (0.0302)	–0.0792 (0.0287)
	*P* value	N/A	<.001	.001	.009	.006
City fixed effect^h^		No	No	No	Yes	Yes
Year fixed effect^i^		No	No	Yes	Yes	Yes
Control^j^		No	Yes	Yes	Yes	Yes
Robust^k^		No	No	No	No	Yes
Observations, N		3600	3600	3600	3600	3600
Values, *R*^2^		0.023	0.035	0.024	0.024	0.024

^a^MM: medical resource mismatch.

^b^BCP: Broadband China Policy.

^c^Gdp: economic level.

^d^N/A: not applicable.

^e^Pop: population structure.

^f^Gov: government intervention.

^g^Urb: urbanization rate.

^h^City fixed effect controls for unobservable factors that vary across cities.

^i^Year fixed effect controls for unobservable factors that vary over time.

^j^Control refers to a set of control variables as mentioned in the previous text.

^k^Robust indicates the use of robust SEs to account for heteroscedasticity.

Column 1 shows the ordinary least squares regression results without any control. The regression coefficient of the BCP on MM is –0.134, which is significant at the 1% level, indicating that the implementation of BCP has reduced the MM by 13.4%. However, it should be noted that this only reflects a correlation. Column 2 adds control variables to control for the impact of other observable factors. The implementation of the BCP has reduced the MM by 13.3%. Column 3 further adds year fixed effects to control for the influence of unobservable factors in different observation years. The result remains unchanged, with only a slight increase in the SE. Column 4 further adds city fixed effects to control for the influence of unobservable factors in the different cities. The implementation of BCP has reduced the MM by 13.2%. Column 5 further uses robust SEs to control for the heteroscedasticity of the error term. The result is the same as before, with the implementation of BCP reducing the MM by 13.2%.

Due to the more complete control of the confounding effects of other factors, the baseline regression result of this study is based on column 5, and hypothesis 1 is clearly supported. The results were similar to those of existing studies [68], which studied the impact of the BCP on urban labor allocation and obtained similar results.

Looking at the control variables in column 5, Gdp reduces the MM by 0.86%, Pop reduces it by 12%, Gov increases it by 13.3%, and Urb reduces it by 7.92%, which is consistent with the previous expectations.

#### Dynamic Test

To conduct a DID regression, the parallel trends assumption must be satisfied between the control group and the experimental group. Otherwise, the regression results may be driven by preexisting differences between the groups. Drawing on the event study methodology commonly used in the field of finance, the following dynamic regression equation is constructed for testing:



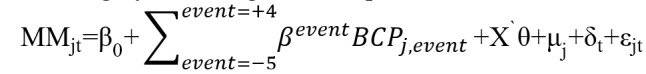



**Figure 1:**
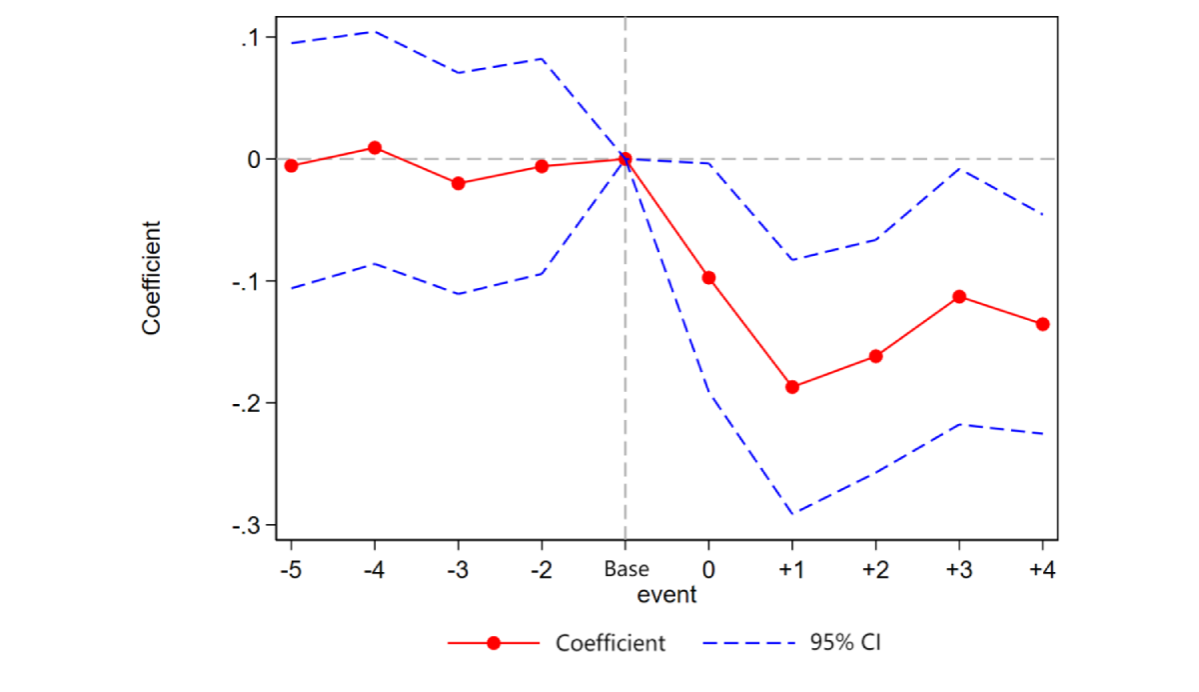
Dynamic test.

In this equation, *event* represents the event period. In the period when the policy is implemented, event is set to 0. In the period before the policy implementation, event is set to –1. In the period after the policy implementation, event is set to +1, and so on for subsequent periods. Following the practice in mainstream literature, event=–1 is chosen as the base period. The interpretation of other variables remains the same and will not be repeated. The specific regression results are shown in Figure 1.

Before the policy implementation (ie, when event ≤–1), the coefficient of the BCP is not statistically significant, indicating that there are no significant preexisting differences between the pilot areas of the BCP and nonpilot areas, thus satisfying the parallel trends assumption. In the periods after the policy shock, the coefficient of BCP is significantly negative and decreases over time before stabilizing. Overall, the BCP has significantly reduced the degree of MM each year.

### Placebo Test

**Figure 2:**
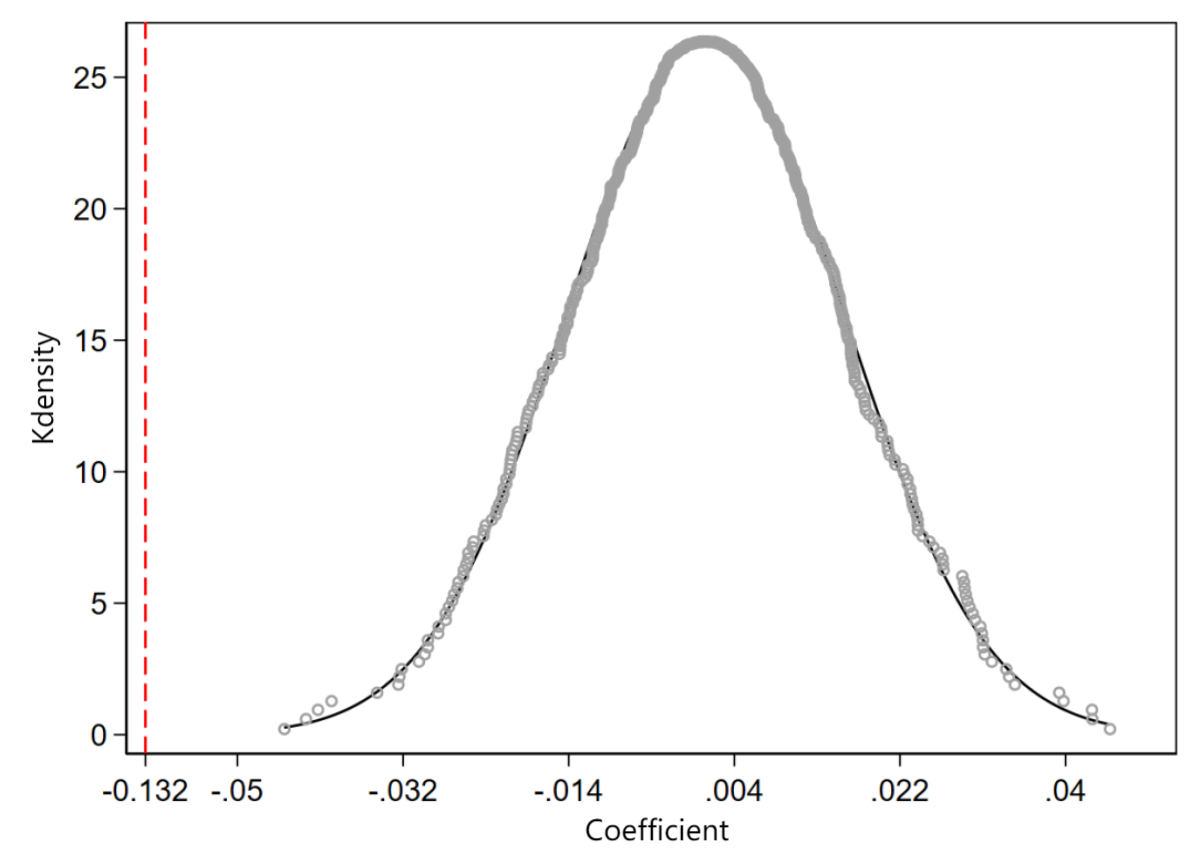
Placebo test.

The baseline regression results of this study may be due to chance grouping rather than a true reflection of the policy effect of BCP. To eliminate the interference of chance factors, we conducted a placebo test, arbitrarily designating a city as a false treatment group and then regressing to obtain a false regression coefficient. This operation was repeated 500 times, and the density of the 500 spurious regression coefficients obtained is shown in Figure 2.

As shown in the figure, the 500 spurious regression coefficients are mostly concentrated around 0 and are significantly different from the real regression coefficient (red dashed line), indicating that the baseline results of this study are not due to chance factors.

### Robustness Analysis

#### Overview

To ensure the robustness of the benchmark regression results, a series of robustness tests were conducted in this section, and the specific results are presented in Table 3.

**Table 3:** Robust regression results.

Variables			MM^a^_solow (column 1)		PSM-DID^b^ (column 2)		IV^c^1 (column 3)		IV2 (column 4)
BCP^d^									
	Coefficient (SE)	–0.218 (0.0737)		–0.123 (0.0208)		–0.183 (0.0395)		–0.119 (0.0273)	
	*P* value	.003		<.001		<.001		<.001	
Kleibergen-Paap^e^			N/A^f^		N/A		32.748		23.946
Cragg-Donald^g^			N/A		N/A		33.661		23.809
City fixed effect^h^			Yes		Yes		Yes		Yes
Year fixed effect^i^			Yes		Yes		Yes		Yes
Control^j^			Yes		Yes		Yes		Yes
Robust^k^			Yes		Yes		Yes		Yes
Observations, N			3600		3600		3600		3600
Values, *R*^2^^l^			0.010		0.023		N/A		N/A

^a^MM: medical resource mismatch.

^b^PSM-DID: propensity score matching difference-in-differences.

^c^IV: instrumental variable.

^d^BCP: Broadband China Policy.

^e^Kleibergen-Paap represents the Kleibergen-Paap rk LM (Lagrange Multiplier) statistic, which performs the underidentification test. The null hypothesis is that the core explanatory variable is not correlated with the IV. The results in the table clearly reject the null hypothesis.

^f^N/A: not applicable.

^g^Cragg-Donald represents the Cragg-Donald Wald F statistic, which performs the weak identification test. The null hypothesis is that the core explanatory variable is weakly correlated with the IV. The results in the table clearly exceed the eigenvalue at the 10% significance level suggested by Stock-Yogo.

^h^City fixed effect controls for unobservable factors that vary across cities.

^i^Year fixed effect controls for unobservable factors that vary over time.

^j^Control refers to a set of control variables as mentioned in the previous text.

^k^Robust indicates the use of robust SEs to account for heteroscedasticity.

^l^As the *R^2^* in the 2-stage IV method does not reflect the true goodness of fit, it is not reported here

#### Replacement of the Dependent Variable

In the previous calculation of the dependent variable MM, the elasticity values of medical labor and medical capital were measured using the Olley-Pakes method. In this section, following the existing studies [69], the Solow residual method was used for recalculation. Simultaneously, the substitution elasticity of urban output was replaced with an upper limit value of 10. The newly obtained dependent variable MM_solow was used, and the specific regression results are shown in column 1: the impact coefficient of the BCP on MM_solow is –0.218, which is significant at the 1% level, indicating that the implementation of BCP has reduced the degree of mismatch of medical resources by 21.8%, and the results remain robust.

#### Propensity Score Matching–DID Method

Considering that the pilot cities for the BCP may not have been arbitrarily selected and that there may be inherent differences between pilot and nonpilot cities, it is precisely these differences that drive the benchmark regression results. To address this concern, this study uses the propensity score matching–DID method to match pilot and nonpilot areas in a 1:1 ratio based on propensity score matching and then conducts a reregression. The results are shown in column 2: the impact coefficient of the BCP on MM is –0.123, which is significant at the 1% level, indicating that the implementation of the BCP has reduced the degree of MM by 12.3%, and the results remain robust. It should be noted that the premise of using the propensity score matching–DID method is that there are no significant differences in covariates between pilot and nonpilot areas. We conducted a covariate balance test; the specific regression results are presented in Table S2 in Multimedia Appendix 1.

#### Instrumental Variable Method

Considering the potential endogeneity issue between the BCP and the degree of MM, it is possible that cities with a lower degree of MM are also cities with higher levels of IT and economic development, making them more likely to be selected as pilot areas for the BCP. In addition, this study may also suffer from omitted variable bias and measurement error issues that could lead to endogeneity. Although the DID approach used in this study partially mitigated the endogeneity problem, to enhance result reliability, we further implemented an instrumental variable (IV) approach to address potential endogeneity issues, following the methodology of a previous study [68].

In reference to existing studies [68], we used the topographic fluctuation of cities as an instrument. On the one hand, cities with larger topographic fluctuation not only increase the cost of broadband construction but also lead to a decrease in the signal quality of broadband networks. Therefore, cities with larger topographic fluctuations face greater difficulties in broadband construction, making them less likely to become pilot cities for the *Broadband China* initiative. On the other hand, topographic fluctuation is a geographical condition with strong exogeneity and does not affect labor allocation distortion. For ease of understanding, we took the reciprocal of topographic fluctuation as IV1. The specific regression results are presented in column 3: the impact coefficient of BCP on MM is –0.133, which is significant at the 1% level, indicating that the implementation of BCP has reduced the degree of MM by 18.3%, and the results remain robust. Furthermore, the results pass the overidentification test (Kleibergen-Paap) and weak identification test (Cragg-Donald).

In reference to existing studies [70], we used the distance from each city to Hangzhou as an instrument. On the one hand, in 2004, the Alibaba Group launched the Alipay service in Hangzhou, which promoted the development of digital finance. Therefore, cities closer to Hangzhou are more likely to have the foundation for digital infrastructure construction and application, making them more likely to be selected as pilot areas for the BCP. On the other hand, this IV is a historical distance variable, satisfying the exogeneity assumption [71,72]. Thus, we obtain IV2. The specific regression results are presented in column 4: the impact coefficient of BCP on MM is –0.119, which is significant at the 1% level, indicating that the implementation of BCP has reduced the degree of MM by 181.9%, and the results remain robust. Furthermore, the results pass the overidentification test (Kleibergen-Paap) and weak identification test (Cragg-Donald).

#### Mechanism Test

To determine which channels the BCP reduces the MM, this study explores and identifies 2 potential channels: the innovation effect and the integration effect. The mechanism test is conducted using a 3-step method, and the specific regression results are presented in Table 4.

**Table 4:** Mechanism test.

Variables		Patent (column 1)	MM^a^ (column 2)	Integration (column 3)	MM (column 4)
BCP^b^					
	Coefficient (SE)	0.225 (0.0807)	–0.127 (0.0238)	0.0786 (0.0365)	–0.126 (0.0236)
	*P* value	.006	<.001	.03	<.001
Patent					
	Coefficient (SE)	N/A^c^	–0.0249 (0.0051)	N/A	N/A
	*P* value	N/A	<.001	N/A	N/A
Integration					
	Coefficient (SE)	N/A	N/A	N/A	–0.0764 (0.0130)
	*P* value	N/A	N/A	N/A	<.001
City fixed effect^d^		Yes	Yes	Yes	Yes
Year fixed effect^e^		Yes	Yes	Yes	Yes
Control^f^		Yes	Yes	Yes	Yes
Robust^g^		Yes	Yes	Yes	Yes
Observations, N		3600	3600	3600	3600
Values, *R*^2^		0.006	0.030	0.007	0.034

^a^MM: medical resource mismatch.

^b^BCP: Broadband China Policy.

^c^N/A: not applicable.

^d^City fixed effect controls for unobservable factors that vary across cities.

^e^Year fixed effect controls for unobservable factors that vary over time.

^f^Control refers to a set of control variables as mentioned in the previous text.

^g^Robust indicates the use of robust SEs to account for heteroscedasticity.

#### Innovation Effect

Research has shown that enhancing broadband infrastructure can elevate urban innovation levels, with a reported increase of 1.94% in innovation for every unit of internet access [73]. It is evident that broadband infrastructure construction plays a crucial role in advancing China’s regional innovation level by directly improving innovation levels and indirectly impacting innovation through the acceleration of human capital accumulation, financial development, and industrial upgrading [74]. The expansion of broadband networks not only broadens the spatial range of network space functions and innovation resource allocation but also offers more opportunities and development space for innovation activities and the evolution of innovation models [75]. Furthermore, there exists a substantial correlation between innovation efficiency and resource allocation efficiency, with higher innovation efficiency leading to enhanced resource allocation efficiency [76]. Hence, it is anticipated that the BCP can alleviate resource mismatches by fostering regional innovation.

In reference to the existing studies [77], this study measures urban innovation levels based on the number of invention patents. Patent numbers are divided into 2 categories: patent applications and patent authorizations. Considering that policy shocks will have an impact on urban innovation levels in the current period and that there is a certain time lag from patent application to authorization, this study uses the number of invention patent applications in each city (Patent) to measure urban innovation levels. The number of city patent applications comes from the China Patent Public Announcement System on the website of the China National Intellectual Property Administration. The regression results are shown in columns 1 and 2: the coefficient of the BCP’s impact on Patent is 0.225, and it is significant at the 1% level, indicating that the implementation of the BCP significantly improves urban innovation levels. The coefficient of Patent’s impact on MM is –0.0249, and it is significant at the 1% level, indicating that the significant improvement in urban innovation levels reduces the mismatch of medical resources, confirming the theoretical expectations.

#### Integration Effect

Market segmentation restricts the free flow of resources. The development of broadband internet can promote market integration and improve resource mismatches [78]. Improving information technology infrastructure construction can achieve resource integration, enhance resource management levels, and improve production efficiency [79]. The internet accelerates production transformation and market integration. It optimizes resource flow between the upstream and downstream through information mechanisms, gradually reducing resource waste and promoting resource growth. In general, the BCP can improve resource mismatches in target areas through market integration [80,81].

Following the methodology of a previous study [82], this study uses *(number of regional hospitals/regional medical expenditure)/(number of national hospitals/national medical expenditure*) to measure regional integration (Integration). The regression results are shown in columns 3 and 4: the coefficient of BCP’s impact on Integration is 0.0786, which is significant at the 5% level. This indicates that the implementation of BCP significantly improves urban integration levels. The coefficient of Integration’s impact on MM is –0.0764, which is significant at the 1% level. This suggests that the significant improvement in integration levels significantly reduces the MM, confirming the theoretical expectations.

### Heterogeneity Analysis

#### Overview

This section tests the heterogeneous hypotheses proposed earlier and conducts an empirical *P* value test for intergroup coefficient differences. The specific regression results are presented in Table 5.

**Table 5:** Heterogeneity results.

Variables			MM^a^				MM				MM		
			Column 1: central and eastern region		Column 2: western region		Column 3: high population size		Column 4: low population size		Column 5: high youth proportion		Column 6: low youth proportion
BCP^b^													
	Coefficient (SE)	–0.159 (0.0292)		–0.0664 (0.0415)		–0.149 (0.0321)		–0.118 (0.0332)		–0.144 (0.0367)		–0.130 (0.0368)	
	*P* value	<.001		.11		<.001		<.001		<.001		<.001	
Empirical *P* value^c^			.002		.002		.008		.008		.016		.016
City fixed effect^d^			No		No		No		Yes		Yes		Yes
Year fixed effect^e^			No		No		Yes		Yes		Yes		Yes
Control^f^			No		Yes		Yes		Yes		Yes		Yes
Robust^g^			No		No		No		No		Yes		Yes
Observations, N			2448		1152		1800		1800		1800		1800
Values, *R*^2^			0.030		0.026		0.037		0.021		0.038		0.024

^a^MM: medical resource mismatch.

^b^BCP: Broadband China Policy.

^c^Empirical *P* value, testing for differences in coefficients between groups, was achieved using a 500-times self-help sampling method.

^d^City fixed effect controls for unobservable factors that vary across cities.

^e^Year fixed effect controls for unobservable factors that vary over time.

^f^Control refers to a set of control variables as mentioned in the previous text.

^g^Robust indicates the use of robust SEs to account for heteroscedasticity.

#### Regional Differences

Hypothesis 2 expects that there are differences in the effects of BCP between different regions, with a larger effect in the central and eastern regions compared with the western region. According to the regional classification standards of the National Bureau of Statistics of China, we classified Sichuan, Yunnan, Guizhou, Tibet, Chongqing, Shaanxi, Gansu, Qinghai, Xinjiang, Ningxia, Inner Mongolia, and Guangxi as the western region, while the rest are classified as the central and eastern regions. The group regression results are shown in columns 1 and 2: the BCP reduces MM by 15.9% in the central and eastern regions, but it does not have a significant impact in the western region. The empirical *P* value indicates a significant difference in the coefficients between the 2 groups. This confirms the expectation of hypothesis 2.

#### Population Size Differences

Hypothesis 3 expects that there are differences in the effects of BCP among different population sizes, with larger cities experiencing a greater effect. On the basis of the median total population at the end of each city, the sample is divided into 2 subsamples: a high population size and a low population size. The group regression results are shown in columns 3 and 4: the BCP reduces medical mismatch by 14.9% in the high population size sample, while it reduces it by 11.8% in the low population size sample. The empirical *P* value indicates a significant difference in the coefficients between the 2 groups. This confirms the expectation of hypothesis 3.

#### Population Structure Differences

Hypothesis 4 expects that there are differences in the effects of BCP among different population structures, with cities having a higher proportion of the young population experiencing a greater effect. Due to the lack of data on the proportion of the young population in the China Urban Statistical Yearbook, considering that the ratio of the young population receiving primary and secondary education is >90% and the ratio of those receiving higher education is close to 60% [83], we use the number of students in ordinary primary, secondary, and higher education institutions as a proxy for the young population. We divide the sample into 2 groups based on the median proportion of these students to the total population: high youth proportion and low youth proportion. The group regression results are shown in columns 5 and 6: the BCP reduces medical mismatch by 14.4% in the high youth proportion sample, while it reduces it by 13% in the low youth proportion sample. The empirical *P* value indicates a significant difference in the coefficients between the 2 groups. This confirms the expectation of hypothesis 4.

## Discussion

### Principal Findings

The theoretical analysis and empirical results of this study demonstrate that the construction of broadband infrastructure significantly reduces the MM by 13.2%. The potential realization channels are innovation effects and integration effects, and this impact is more pronounced in the central and eastern regions, cities with large populations, and cities with a high proportion of young people.

### Limitations

This study still has some limitations. For example, how to establish a mathematical model to accurately depict the quantitative relationship between the construction of broadband infrastructure and the MM from a mathematical perspective and incorporate mechanism analysis and heterogeneous analysis into this model, ultimately forming a systematic theoretical analysis framework, is worth exploring in the future.

### Conclusions

This study proposes 4 policy recommendations. First, there is a need to promote the implementation of BCP more extensively. The benchmark regression results demonstrate that the BCP significantly reduces the mismatch of health care resources. This includes the expansion of BCP to develop information technology in more cities, enhancing the integrated use of broadband infrastructure in the health care service industry, and a comprehensive transformation of the health care service industry in various aspects. Furthermore, the digitization of health care construction and the development of information-based health services such as electronic health record systems, telemedicine services, and mobile health apps are imperative. Enhancing broadband infrastructure construction and using it as a precursor to improving the mechanism of health care resource allocation is crucial. This will strengthen broadband internet access in the health care industry, promote efficient resource exchange among health care institutions, optimize resource allocation using technologies such as big data and artificial intelligence, and reduce the mismatch of health care resources [84].

Second, there is a need to focus on the level of technological innovation and resource integration capacity. The mechanism test indicates that the BCP reduces the MM by enhancing the level of innovation and resource integration capacity. This entails accelerating the deployment of emerging broadband infrastructure, focusing on the coordination between broadband infrastructure and innovation policy and capacity, establishing interdisciplinary innovation centers, and promoting collaborative innovation among the health care, IT, and communication fields. Moreover, broadening and deepening the integration of broadband infrastructure and medical resources to guide better integration and allocation of interregional medical resources is essential. This can be achieved through the establishment of a unified resource management and scheduling platform [85].

Third, the prioritization of the BCP implementation in the central and eastern regions, cities with large populations, or regions with a high proportion of young people is recommended. The results of the heterogeneous regression indicate that the impact of BCP is more significant in these cities. This includes leveraging the leading role of key cities, reinforcing their role as leaders in health care information and broadband infrastructure, and capitalizing on their demonstration effect and radiation. Simultaneously, under the current conditions, it is crucial to introduce social capital, including demand, technology, and funds, to assist cities with relatively backward broadband infrastructure in overcoming their disadvantages and catching up with advanced cities [86].

Finally, macrocontrol should be strengthened to encourage factor mobility. Owing to differences in economic levels and support, there is a substantial gap in the allocation of medical resources in China, primarily due to the lack of medical factor flow. It is imperative to strengthen the macrocontrol and coordination capacity, formulate overall medical resource planning, clarify the objectives and development direction of medical resource allocation in various regions, improve the resource allocation system, and enhance the macrocontrol of the medical and health industries. Encouraging the flow of medical resource elements and increasing policy flexibility will help adapt to rapid changes in medical needs and technological development trends and better satisfy the medical needs of different levels [87].

## Appendix

Multimedia Appendix 1 Supplemental tables.

## Abbreviations

BCP: Broadband China Policy
DID: difference-in-differences
GDP: gross domestic product
IV: instrumental variable
MM: medical resource mismatch
