# Supplementary material for: The Impact of Broadband Infrastructure Construction on Medical Resource Mismatch: Quasi-Natural Experiment From the Broadband China Policy
Source: J Med Internet Res. 2024 Mar 21;26:e53921. doi: 10.2196/53921 (PMC10995788; doi:10.2196/53921)
Supplement: Multimedia Appendix 1 [file jmir_v26i1e53921_app1.docx]

# Multimedia Appendix 1

Table S1. Definition of variables

| Variables | Name | Definition |
| --- | --- | --- |
| MM | Mismatch of Medical Resources | Drawing on Hsieh & Klenow's (2009) [59] methodology, as described above |
| BCP | Broadband China Policy | A city is assigned a value of 1 for the year the policy is implemented and beyond, and 0 for the rest |
| Gdp | Economic Level | Log(1 + per capita GDP) |
| Gov | Government Intervention | Government fiscal expenditure/regional GDP |
| Pop | Population Structure | Average number of employees/average total population |
| Urb | Urbanization Rate | Non-agricultural population/total population |

Table S2. Covariate equilibrium test

| Variable | Mean | |  | t-test | |
| --- | --- | --- | --- | --- | --- |
|  | Treated | Control | %bias | t | P>**\|** t **\|** |
| Gdp1 | 2.5282 | 2.4782 | 3.4 | 0.68 | 0.499 |
| Pop1 | 0.55706 | 0.55688 | 0.1 | 0.02 | 0.985 |
| Gov1 | 0.26599 | 0.26891 | -2.4 | -0.48 | 0.633 |
| Urb1 | 0.4555 | 0.46473 | -4.6 | -0.89 | 0.374 |
